# Reverse shoulder arthroplasty for proximal humerus fracture: does tuberosity reinsertion improve the functional outcomes more than 5 years after surgery?

**DOI:** 10.1007/s00590-025-04227-2

**Published:** 2025-03-19

**Authors:** Noémie Allio, Thomas Amouyel, Marc Saab, Christophe Chantelot

**Affiliations:** 1 CHU Lille Hôpital Roger Salengro, Lille, Nord (59), France; 2 Université Médecine Lille Henri Warembourg, Lille, Nord (59), France; 3 Université de Lille, CNRS, INSERM, CHU Lille, UMR9020-U1277—CANTHER—Cancer Heterogeneity Plasticity and Resistance to Therapies, Lille, Nord (59), France

**Keywords:** Reverse shoulder arthroplasty, Tuberosities, Constant score, Geriatric evaluation

## Abstract

**Introduction:**

While tuberosity reinsertion significantly improves the short-term functional outcomes of reverse shoulder arthroplasty done for proximal humerus fracture, we do not know how well these results hold over the long term. The objective of this study was to analyze the effect of tuberosity reinsertion on the quality of life of patients and the functional outcomes of the operated limb after a minimum follow-up of 5 years.

**Methods:**

Sixty-two patients were included. Their mean age at the final review was 79 ± 10 years. The Katz and Lawton scales, Constant score, DASH and SSV were collected. Radiographs were made at the final assessment to analyze the position of the tuberosities and to look for radiological signs of implant loosening.

**Results:**

The mean follow-up was 6.7 ± 1.5 years. The tuberosities had been reinserted in 35 patients (56%). There were no statistically significant differences between groups in the Katz (*p* = 0.60) and Lawton (*p* = 0.49) scales, nor the DASH (*p* = 0.45) or SSV (*p* = 0.49) at the final review. The Constant score was significantly better in the patients who had their tuberosities reinserted (*p* = 0.01), also the active forward flexion (*p* = 0.02), the internal rotation (*p* = 0.01), and the external rotation arm abduction (*p* = 0.02), but there was no significant difference for external rotation elbow at side (*p* = 0.14). None of the patients underwent revision surgery for implant loosening.

**Conclusion:**

Tuberosity reinsertion has a functional benefit beyond 5 years postoperative, although it does not appear to have a significant effect on the geriatric outcomes or the subjective clinical scores. The patients regained satisfactory independence for an orthogeriatric population.

**Level of evidence:**

Level IV—Retrospective study.

## Introduction

Proximal humerus fractures (PHF) make up 6% of all fractures in adults and 10% of fractures after 65 years of age [1]. The humerus is the third most common fracture location in older adults, after the proximal femur and distal radius [2]. The incidence of PHF in France is 30,000 cases per year [1]. Reverse shoulder arthroplasty (RSA) has better clinical outcomes [3] and a lower revision rate than hemiarthroplasty for the treatment of displaced PHF [4]. Grammont and Baulot both suggested that the action of the deltoid muscle on the RSA was sufficient to compensate for the lack of rotator cuff muscles, especially on the recovery of forward flexion and abduction [5]. In the early studies by Cazeneuve [6] and Gallinet [7], reinserting the tuberosities was not recommended. However, more recent studies have found a significant improvement in the Constant score when the tuberosities are reinserted (60–79%) versus not reinserted (50–56%) [8–13]. But these studies did not report the effect of tuberosity reinsertion on the patient’s quality of life or independence [10, 12]. Also, very few studies have investigated the functional benefits of tuberosity reinsertion in the long term [11].

The primary objective of our study was to determine if tuberosity reinsertion improves shoulder function in the long term. The secondary objectives were to determine the consequences on the patients’ quality of life, determine the condition of the tuberosities on radiographs and implant survival. The null hypothesis was that there is no significant difference in the Constant score more than 5 years after RSA whether or not the tuberosities are reinserted.

## Methods

### Patients

This was a retrospective, single-center, comparative, multisurgeon cohort study. Five surgeons were involved (one level 4, threeseven level 3 and five level 2 according Tang and Giddins [14]).

**Fig. 1:**
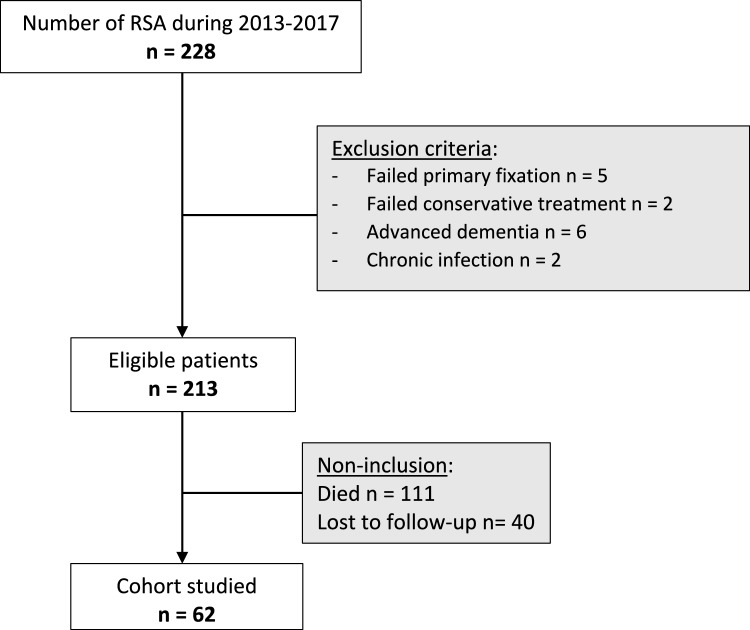
Flow chart

Eligible were patients with a Neer 3 or 4 PHF [15] treated by RSA between January 2013 and December 2017 and who had a minimum follow-up of 5 years (n = 228). After applying the exclusion criteria (Fig. 1), 213 patient records were reviewed. Of these, 111 patients had died, and 40 patients were lost to follow-up.

The remaining 62 patients were invited for a final in-person review more than 5 years after RSA to carry out a full clinical examination of the shoulder and to make follow-up radiographs. All patients provided their written consent after having received clear, accurate information about the study. This study was approved by our facility’s data protection officer (Ref number DEC22-302).

### Surgical technique

A cemented UNIC® RSA implant (EVOLUTIS™) was used in all patients. It consists of a 30-mm diameter titanium glenoid base secured with two to four standard cancellous bone screws (5-mm diameter), a 38-mm diameter stainless steel glenosphere, a cemented highly polished titanium alloy humeral stem with 20° retroversion and a polyethylene insert. The RSA was implanted using a superolateral surgical approach [16]. Given the lack of consensus about the benefits of tuberosity reinsertion when these surgeries were done in 2013–2017, the decision to perform reinsertion was left up to the surgeon, based on intraoperative findings and preferences.

After exposing the fracture site and the rotator cuff, any bone fragments without periosteum were excised. The greater and lesser tuberosities were separated using bone scissors in Neer 3 fractures. The tuberosities were captured with two to four Mersuture® #1, PDS II® loop, or Vicryl Plus® #0 sutures. The surgeon was free to do tenodesis or tenotomy with the tendon of the long head of biceps. After glenoid exposure and labrum excision, a threaded guide pin was inserted into the middle of the glenoid, which was then prepared with a motorized burr.

Before cementing the chosen humeral stem, two Mersuture® #1 sutures were positioned in the lateral cortex of the humeral shaft to set the stage for reinserting the tuberosities. Once this was completed, the tuberosities were reinserted using the tension-band wiring technique described by Boileau et al. [17].

The operated limb was immobilized for 3 weeks in a sling. Passive and active-assisted mobilization of the shoulder was initiated in the immediate post-operative period. Active mobilization was initiated after weaning of the sling.

### Clinical and radiological assessment

A full clinical examination of the shoulder was done by a surgeon (NA) who was not involved in the RSA implantation. The Constant [18, 19], the Disabilities of the Arm, Shoulder and Hand (DASH) and the Subjective Shoulder Value (SSV) were collected. We documented active range of motion, measured with a goniometer for the forward flexion, abduction and external rotation. The internal rotation (IR) was measured as the highest vertebral level the patient could reach in the back, then conversed into numerical values as in the Constant score.

The Activities of Daily Living (ADL) and instrumental Activities of Daily Living (iADL) scales were completed to determine the patient’s independence and their overall functional prognosis after the surgery.

**Fig. 2:**
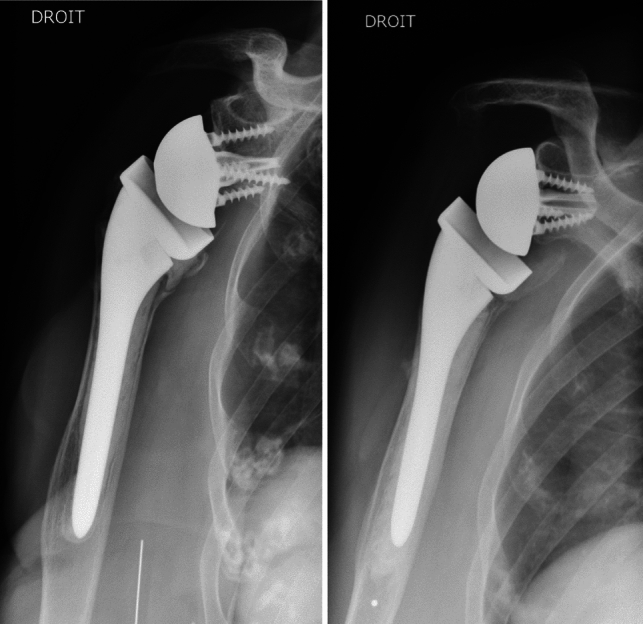
Postoperative radiographs: tuberosities reinserted (left image) or not reinserted (right image)

Conventional radiographs of the operated shoulder were made at the final assessment (AP views in three different rotations and Lamy lateral view) to determine fracture union and the position of the tuberosities if they had been reinserted (Fig. 2). The tuberosities were considered healed if the greater tuberosity was visibly continuous with the humeral shaft on the AP view in neutral rotation. The tuberosities’ position and potential secondary osteolysis were determined on the AP views (all three rotations) and the lateral view [20]. We looked for the presence of periprosthetic radiolucent lines (≥ 2 mm) [21] and notching (Sirvaux classification) [22] suggestive of implant loosening.

### Statistical analysis

Data were analyzed statistically using SPSS® software (Version 20.0, SPSS IBM, New York, U.S.A). The Shapiro–Wilk test was used to determine if the data was normally distributed. Student’s *t* test was used to compare the means of normally distributed continuous variables. A non-parametric Mann–Whitney was used to compare continuous variables that were not normally distributed. Due to the small sample size, Fisher’s exact test was used to test hypotheses involving nominative or ordinal parameters. The null hypothesis was rejected when *p* < 0.05.

## Results

### Clinical outcomes

The tuberosities had been reinserted in 35 patients (56%). The mean follow-up was 7.2 ± 1.4 years in patients whose tuberosities had not been reinserted (group 1) and 6.5 ± 1.3 years in patients whose tuberosities had been reinserted (group 2) (*p* = *0.07*). The general characteristics of the patients were comparable between the two groups. (Table 1) The Constant (absolute and adjusted) was significantly better in the group with tuberosity reinsertion (absolute Constant of 69.8 ± 17.1) than in the group without reinsertion (56.7 ± 19.7) (*p* = *0.01*). There was a statistically significant difference in favor of reinsertion for external rotation arm abduction (ER2) (*p* = *0.02*), internal rotation (*p* = *0.01*), active forward flexion (*p* = *0.02*) and active abduction (*p* = *0.04*). There was no significant difference for external rotation elbow at side (ER1) (*p* = *0.14*), DASH (*p* = *0.45*) or SSV (*p* = *0.49*) (Table 2).

**Table 1:** Patient demographics

		Total (n = 62)	Group 1 Tuberosities not reinserted (n = 27)	Group 2 Tuberosities reinserted (n = 35)	*p*
Follow-up (years) mean ± SD		6.7 ± 1.5	7.2 ± 1.4	6.5 ± 1.3	0.07
Age at surgery (years) mean ± SD		73 ± 10	74 ± 10	71 ± 9	0.17
Age at last follow-up (years) mean ± SD		79 ± 10	82 ± 10	78 ± 9	0.08
Females n (%)		50 (80.6%)	93%	71%	
BMI (mean ± SD)		28.1 ± 5.4	28.3 ± 5.8	27.8 ± 5.2	0.72
Smoker, n (%)		5 (8.1%)	1 (3.7%)	4 (11.4%)	0.38
Alcohol, n (%)		10 (16.1%)	5 (19%)	5 (14%)	0.74
Dominant arm operated on n (%)		35 (56.5%)	17 (63%)	18 (51.4%)	0.36
Fracture context n (%)	Low energy	54 (87.1%)	25 (92.6%)	29 (83%)	0.45
	High energy	8 (12.9%)	2 (7.4%)	6 (17%)	
Living situation n (%)	Home	41 (66.1%)	18 (66.7%)	23 (65.7%)	0.94
	At home with assistance	8 (12.9%)	3 (11.1%)	5 (14.3%)	
	Nursing home	13 (21%)	6 (22.2%)	7 (20%)	
Neer classification n (%)	Neer 3	25 (40%)	11 (40.7%)	14 (40%)	0.95
	Neer 4	37 (60%)	16 (59.3%)	21 (60%)	
Time to surgery (days) mean ± SD		2.1 ± 3	2 ± 3	2 ± 3	0.95
Length of hospital stay (days) mean ± SD		6.4 ± 4.5	6.9 ± 4.4	6 ± 4.6	0.48
ASA score 3 or 4 n (%)	1	4 (6.5%)	1 (3.7%)	3 (8.6%)	0.9
	2	23 (37.1%)	10 (37%)	13 (37.1%)	
	3	29 (46.8%)	13 (48.1%)	16 (45.7%)	
	4	6 (9.6%)	3 (11.1%)	3 (8.6%)	
Complications, n (%)	Glenoid loosening	1 (1.6%)	0 (0%)	1 (2.9%)	0.4
	Periprosthetic fracture	2 (3.2%)	1 (3.7%)	1 (2.9%)	
	Dislocation	2 (3.2%)	2 (7.4%)	0 (0%)

**Table 2:** Functional and clinical outcomes at last follow-up

	Mean ± SD n = 62	Tuberosities not reinserted n = 27	Tuberosities reinserted n = 35	*p*
Constant-Murley score (/100)	64.1 ± 19.2	56.7 ± 19.7	69.8 ± 17.1	0.01
Adjusted Constant score (/100)	64.2 ± 19	64.1 ± 19.4	77.1 ± 16.5	< 0.01
AFF (°)	116 ± 32	105 ± 32	125 ± 31	0.02
Abduction (°)	99 ± 26	92 ± 25	105 ± 25	0.04
ER1 (°)	47 ± 24	42 ± 22	51 ± 25	0.14
ER2 (°)	38 ± 23	30 ± 20	44 ± 24	0.02
IR (°)	2 ± 1	2 ± 1	3 ± 1	0.01
DASH (%)	41.7 ± 22.0	44.6 ± 22.0	34.1 ± 15.5	0.45
SSV (%)	69.0 ± 25.4	66.1 ± 27.6	70.9 ± 24.0	0.49
ADL (/6 points)	4.8 ± 1.6	4.7 ± 1.7	4.9 ± 1.6	0.60
iADL (/8 points)	5 ± 2.9	4.7 ± 2.9	5.2 ± 2.9	0.49

AFF, active forward flexion; ER1, external rotation arm at side; ER2, external rotation in 90° abduction; IR, internal rotation; DASH, Disabilities arm shoulder and hand; SSV, subjective shoulder value; ADL, activities of daily living; iADL, instrumental activities of daily living

There was no significant difference between groups in the ADL (*p* = *0.60*) and iADL (*p* = *0.49*).

### Radiological outcomes

**Fig. 3:**
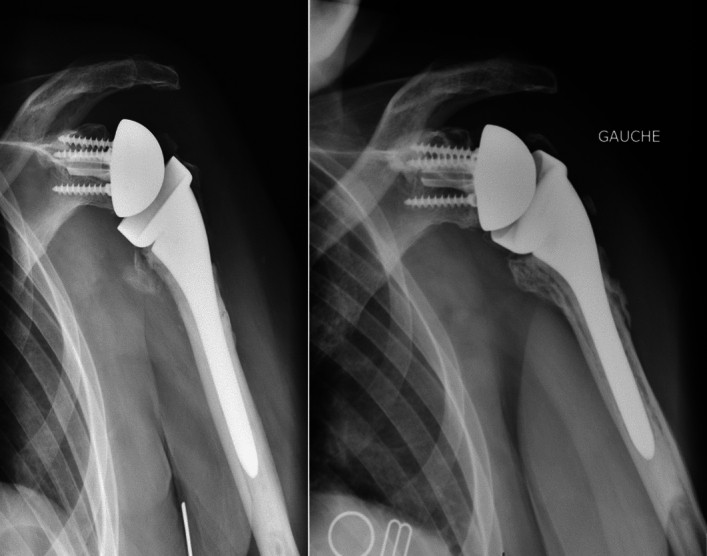
AP radiographs of a left shoulder showing osteolysis of the tuberosities: 1-month radiograph (left image) and 5-year radiograph (right image) showing greater tuberosity osteolysis

**Fig. 4:**
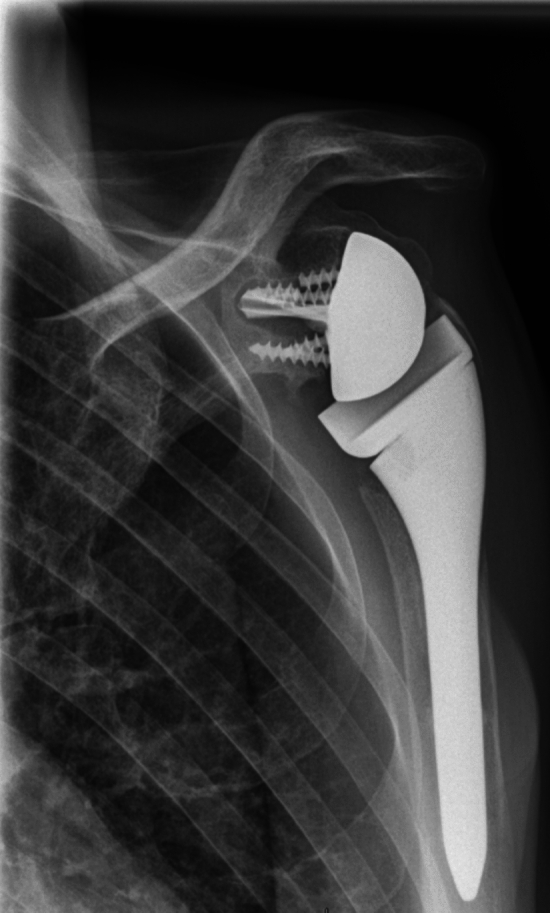
Loosening of glenoid implant

Among the 35 patients in group 2, 13 had secondary lysis of the tuberosities visible on the final radiographs (Fig. 3). One patient in group 2 had glenoid implant loosening associated with fracture of the metaglenoid screw 5 years after RSA implantation (Fig. 4), with a periprosthetic radiolucent line visible. We found the glenosphere to be too high, leading to notching (grade 1), which may have been the cause of this loosening [22]. This patient chose not to undergo revision surgery.

### Complications

**Fig. 5:**
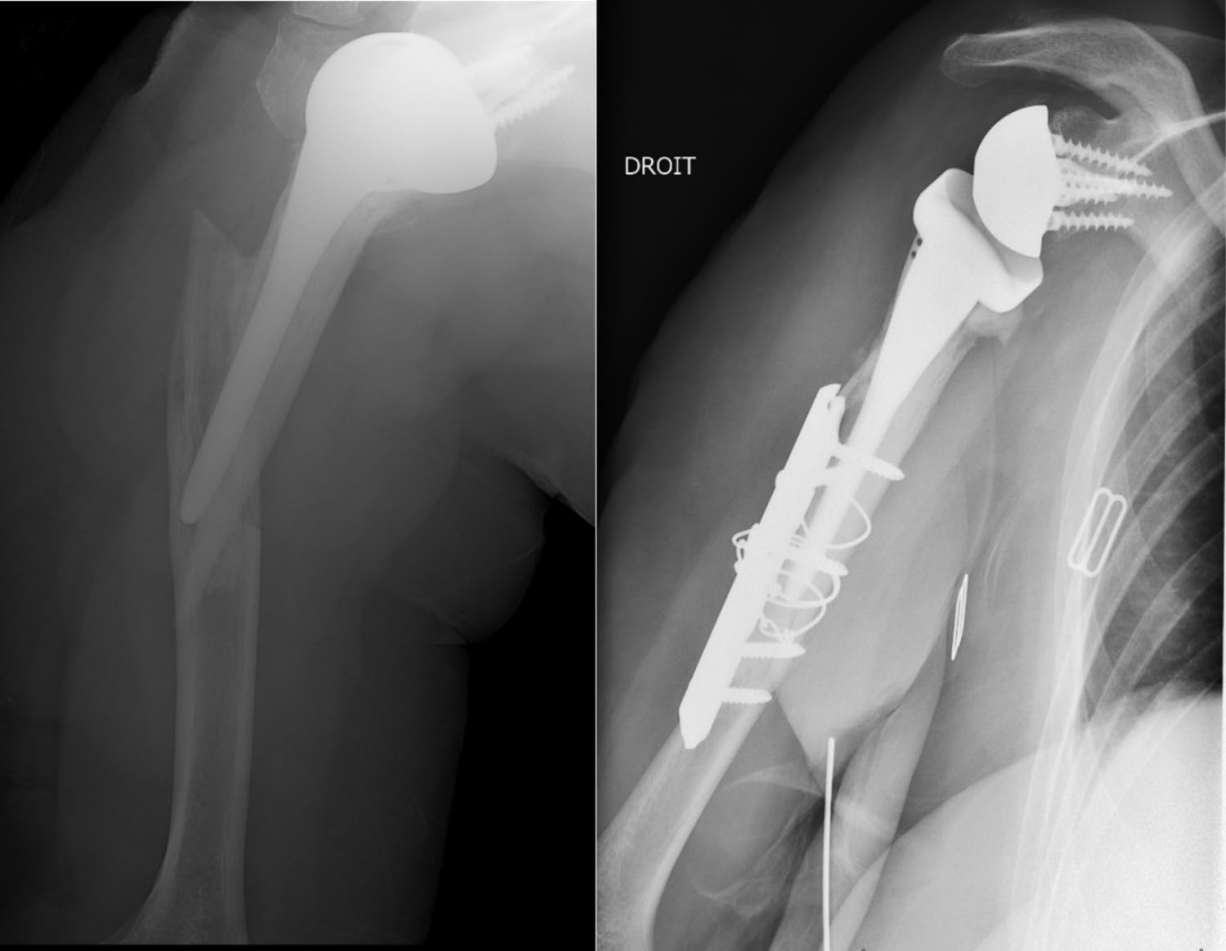
Periprosthetic fracture treated by plate fixation with cerclage wire

Two patients had a type A periprosthetic fracture in the Wright & Cofield classification [23]: 1 patient in group 1 at 2 months postoperative and 1 patient in group 2 at 4 years postoperative. In both cases, a new surgical intervention was done for plate fixation, without changing the RSA implants. (Fig. 5).

Two patients in group 1 had a dislocation episode within the first 3 months after the RSA was implanted. Revision surgery was done to change the polyethylene insert.

## Discussion

Reinserting the tuberosities during RSA significantly improves the Constant score (absolute and adjusted) after more than 5 years’ follow-up (*p* = *0.01*). It also improves active forward flexion (*p* = *0.02*), ER2 (*p* = *0.02*), IR (*p* = *0.01*) and active abduction (*p* = *0.04*). These findings are similar to shorter term studies, which found a statistically significant improvement in the Constant score and recovery of joint range of motion when the tuberosities are reinserted [8–10, 12, 13]. However, we found no significant effect on ER1 (42° ± 22° without reinsertion versus 51° ± 25° with reinsertion; *p* = *0.14*), contrary to the studies by Gallinet et al. (*p* = *0.0015*) [10], Ohl et al. (*p* < *0.001*) [8] and Derksen et al. (*p* = *0.33*) [13]. (Table 3) Nevertheless, the improved functional scores and mobility were long-lasting in the patients with PHF who underwent tuberosity reinsertion during RSA treatment.

**Table 3:** Comparison with previous studies

Study/Year	Follow-up (years, mean, range)	Number of patients	Absolute Constant (mean)		Forward flexion (degrees, mean)		ER1 (degrees, mean)		IR (numerical value, mean)		SSV (mean)		DASH (mean)	
			*GT* +	*GT–*	*GT* +	*GT–*	*GT* +	*GT–*	*GT* +	*GT–*	*GT* +	*GT–*	*GT* +	*GT–*
Gallinet et al. [15] 2013	2 (1.1–5.1)	41	60.1	51.7	117.1	95.7	14.8	0	3	2	NR	NR	31.5	39.8
Ohl et al. [13] 2018	2.3 (1–5)	420	61	53.2	126.7	100.6	22	6.6	4.8	4	75.5	56.5	NR	NR
Gallinet and Cazeneuve [16] 2019	7.6 (5–19)	119	60	50	126	100	21	6	4.4	3	73	6	NR	NR
Luciani et al. [17] 2019	5 (5–5.6)	55	72.5	52.2	135	108	28	5.7	3	1	NR	NR	16.8	37.4
Barros et al. [14] 2020	4.8 (2.7–7.3)	28	79	55	135	90	60	30	NR	NR	NR	NR	NR	NR
Derksen et al. [15] 2024	1 (0.9–1.3)	50	71.3	56.3	NR	NR	24.9	14	NR	NR	82.7	68	18.4	31.9
Our study	6.7 (5–10)	62	69.8	56.7	124.7	105.4	50.9	41.9	3	2	70.9	66.1	34.1	44.6

GT + : tuberosities repaired; GT–: tuberosities not repaired, NR: not reported; ER1, external rotation arm at side; IR, internal rotation; DASH, Disabilities arm shoulder and hand; SSV, subjective shoulder value

There was no significant difference between groups in the DASH (*p* = *0.45*) and SSV (*p* = *0.49*), although both scores were better in the patients in group 2. (Table 2) Ohl et al. found a statistically significant improvement in the SSV at 2 years’ follow-up in patients whose tuberosities were not reinserted (*p* < *0.001*) [8] (Table 3), as did Derksen et al. in a cohort of 50 patients with a mean follow-up of 1 year (*p* = *0.016*) [13]. Luciani et al. found a significantly lower DASH when the tuberosities were reinserted (*p* = *0.003*) [12]. However, Gallinet et al. found no difference in the DASH score in their case series of 41 patients (*p* = *0.14*) [10] (Table 3). The mean age was similar in these studies, ranging from 76.7 to 78.5 years [8–12]. In our study, the mean age was comparable between groups (82 ± 10 years – group 1; 78 ± 9 years – group 2) at the final review. (Table 1) We assume that the functional demands were less given that patients were older when re-evaluated 5 or more years later.

No matter if the tuberosities were reinserted or not, the RSA procedure allowed the patients to maintain satisfactory independence. There were no significant differences in the geriatric evaluation, with a similar ADL score between the two groups (4.7 ± 1.7 group 1 versus 4.9 ± 1.6 group 2, *p* = *0.60*) and a slightly higher iADL score in group 2 (4.7 ± 2.9 group 1 versus 5.2 ± 2.9 group 2, *p* = *0.49*). This can be explained by the somewhat diminished independence in this orthogeriatric population, with 13 patients (21%) residing in a nursing home and 8 patients (13%) receiving home aid, evidence of some loss of independence.

At the final review, secondary osteolysis was found in 13 of the 35 patients whose tuberosities were reinserted. We did not have enough patients in this study for a subgroup analysis. Papadopoulos et al. found a significant improvement in the Constant score when the tuberosities had healed versus when they had not (*p* < *0.001*) [24]. Gunst et al. also found a significant clinical improvement in the Constant score (*p* = *0.04*), forward flexion (*p* = *0.04*) and external rotation (*p* = *0.01*) when the greater tuberosity had healed compared to secondary lysis [25].

No revision surgery was done in our cohort for implant loosening. The single patient who had loosening of the glenoid implant did not want to be re-operated. Two patients had a periprosthetic fracture of the humeral shaft and required another surgery to fix the fracture; their implants did not need to be changed. Maugendre et al. mentioned the low functional demands in these patients who did not survive long enough to wear out the implants [26]. Two patients in group 1 had a dislocation within 3 months of the RSA implantation. Not reinserting the tuberosities may have contributed to this instability. Gallinet and Cazeneuve suggested that the only true factor responsible for instability was excision of the tuberosities (*p* < *0.0001*) [11].

The current study has several strengths. All patients were operated on using the same surgical technique and the same implants. Few studies have reported a long-term follow-up [11, 12], with a mean follow-up of 6.7 ± 1.2 years in our study. Our study is also the only one to have done a geriatric assessment, finding no consequences on patient independence whether or not the tuberosities were reinserted.

The limitations of our study are its retrospective and single-center design, high mortality rate of 52% (Fig. 1), which is attributed to the patients’ advanced age and the follow-up being more than 5 years after the RSA implantation. The multisurgeon nature may also have reduced the comparability of the groups. A comparative analysis of patients who developed secondary osteolysis of the tuberosities would have provided a better comparison with pre-existing studies. Also, a CT scan would have provided a more thorough analysis of tuberosity healing and easier detection of loosening compared to radiographs [27]. However, a CT scan is more complex and less ethical in an orthogeriatric population who may be hesitant to undergo additional diagnostic tests.

## Conclusion

Tuberosity reinsertion when implanting RSA for PHF significantly improves the Constant score (absolute and adjusted) and the shoulder’s mobility at more than 5 years’ follow-up. We found no significant difference in the Katz and Lawton geriatric scales, nor in the DASH or SSV scores. The patients regained satisfactory independence given that this was an orthogeriatric population with lower functional demands.

## Data Availability

No datasets were generated or analysed during the current study.

## Declarations

### Generative AI and AI-assisted technologies in the writing process

No artificial intelligence software was used.
